# WNT-pathway components as predictive markers useful for diagnosis, prevention and therapy in inflammatory bowel disease and sporadic colorectal cancer

**DOI:** 10.18632/oncotarget.1571

**Published:** 2014-01-11

**Authors:** Annalucia Serafino, Noemi Moroni, Manuela Zonfrillo, Federica Andreola, Luana Mercuri, Giuseppe Nicotera, Joseph Nunziata, Riccardo Ricci, Armando Antinori, Guido Rasi, Pasquale Pierimarchi

**Affiliations:** ^1^ Institute of Translational Pharmacology, National Research Council, Rome, Italy; ^2^ Department of Surgery, Catholic University of Rome, Italy; ^3^ Department of Pathology, Catholic University of Rome, Italy; ^4^ Department of Experimental Medicine and Biochemical Science, University of Rome “Tor Vergata”, Italy; ^5^ European Medicines Agency, London, United Kingdom

**Keywords:** Wnt/β-catenin pathway, Colorectal carcinogenesis, Inflammatory Bowel Disease, Diagnostic/therapeutic biomarkers, Multiparametric analysis

## Abstract

The key role of the Wnt/β-catenin signaling in colorectal cancer (CRC) insurgence and progression is now recognized and several therapeutic strategies targeting this pathway are currently in developing. Wnt/β-catenin signaling not only dominates the early stages of sporadic colorectal cancer (SCC), but could also represent the connection between inflammatory bowel diseases (IBD) and increased risk of developing SCC. The knowledge on the sequential molecular events of Wnt-signaling cascade in IBD and during colorectal carcinogenesis, might provide new diagnostic/prognostic markers and could be helpful for optimizing the treatment protocols, thus improving the efficacy of Wnt-targeting therapies. We performed a comparative evaluation of the expression of some crucial molecules participating to Wnt signaling in an animal model of chemically-induced CRC and in human tissues obtained from patients suffering from IBD or at sequential stages of SCC. Specifically, we analyzed upstream events of Wnt signaling including β-catenin nuclear translocation and loss of E-cadherin and APC functions, and downstream events including c-Myc and Cyclin-D1 expression. We demonstrated that these crucial components of the Wnt/β-catenin pathway, when evaluated by immunohistochemistry using a multiparametric approach that includes the analyses of both expression and localization, could be potent markers for diagnosis, prevention and therapy in IBD and SCC, also possessing a predictive value for responsiveness to Wnt-targeting therapies. Furthermore, we showed that the animal model of chemically-induced CRC mimics the molecular events of Wnt signaling during IBD and SCC development in humans and may therefore be suitable for testing chemopreventive or therapeutic drugs targeting this pathway.

## INTRODUCTION

Colorectal cancer (CRC) is the second leading cause of cancer-related deaths in the developed countries [1], where it has a leading position in malignant cancer-associated morbidity and mortality. Liver metastases occur in 35–50% of patients [2], and once they have developed, the prognosis is poor, with an expected median survival time of 6–9 months for untreated disease [3]. Although there have been advances in radiotherapy, chemotherapy, and immunotherapy, surgical excision of the localized disease is currently the only means of improving survival in these patients [4-6]. Even if the addition of new chemotherapeutic agents such as Irinotecan and Oxaliplatin to standard 5-Fluorouracil-based chemotherapy improved the survival of patients with metastatic CRC to about 20 months [7-10], the CRC-derived metastatic disease, and particularly peritoneal carcinomatosis, is basically considered by oncologists a terminal condition for which systemic chemotherapy results almost inefficient. The lack of effective and well-tolerated treatments for advanced CRC highlights the need for innovative approaches for diagnosis, prognosis and therapy for colorectal cancer. Furthermore, chemoprevention is an attractive emerging option to reduce CRC mortality and researches aiming to develop natural or synthetic chemopreventive agents are giving promising results [11].

In the last years, great attention has been given to the so called biological therapy, including innovative therapies employing cells, monoclonal antibodies or specific inhibitors of important steps of cell proliferation and transformation. These therapies are based on the knowledge of molecular pathways involved during cancer development and on the discovery of new specific molecular targets. Drugs directly acting on components of the signaling pathways implicated in tumorigenesis have exhibited clinical benefit in patients with various tumor types, including CRC [12-14].

Therefore, deepening of knowledge on the molecular pathways actively involved in CRC insurgence and progression could potentially provide new targets for drug delivery and therapy, allowing to overcome the poor response to the current therapeutic strategies. Crucial molecules of these signaling should be also validate as new CRC-related biomarkers for the improvement of the current diagnostic/prognostic tools. Moreover, it is recognized that CRC represents a complication of inflammatory bowel diseases (IBD) and patients with long-standing IBD have an increased risk of developing CRC. Many of the molecular alterations responsible for sporadic colorectal cancer (SCC) also seem to play a role in colitis-associated colon carcinogenesis [15]. Thus, knowledge of signaling pathways activated in IBD could also be crucial for the identification of new biomarkers useful for preventive targeted therapy and for the early detection of CRC in patients suffering for Chron's disease or ulcerative rectocolitis.

In this context, the possibility to induce colon tumors in animals provides the opportunity to study various aspects of the carcinogenetic process, including detailed information on the subsequent events leading to the formation of neoplastic lesions. In particular, animal models of CRC could be useful to analyze the expression of novel markers of tumor development during colorectal cancer insurgence, progression and metastases and to identify possible targets for new chemopreventive and/or therapeutic drugs.

In the last decade, numerous studies demonstrated the key role of deregulation or constitutive activation of the Wnt pathway in the initiation and progression of different forms of human cancer, including CRC [16-18]. Hence, several molecular components in the Wnt signaling have been proposed as novel targets for cancer therapy [17,19, 20]. In non-transformed cells, β-catenin, the most important mediators of the Wnt signaling [17, 19], exists in a cadherin-bound form that regulates cell-cell adhesion, and the β-catenin excess, not segregated by E-cadherin on the cell membrane, is rapidly phosphorylated by glycogen synthetase kinase-3β (GSK-3β) in the adenomatous polyposis coli (APC)/axin/GSK-3β destruction complex and is subsequently degraded by the ubiquitin-proteosome pathway. In tumor cells, Wnt signaling causes, by inactivating the APC/axin/GSK-3β complex, ß-catenin accumulation in the cytosol and its translocation into the nucleus. Nuclear β-catenin acts as a co-activator for TCF/LEF-mediated transcription and ultimately modulates cell proliferation, survival and differentiation. Inactivating mutations of APC or stabilizing mutations of β-catenin, leading to constitutive activation of the Wnt/β-catenin pathway, have been recovered in various cancers, including CRC [16, 17, 21]. It has been recently reported that Wnt-pathway activation not only dominates the early stages of sporadic CRC, but is also involved in IBD-associated carcinogenesis [22]. Drugs used in the clinical practice for IBD treatment, such as the aminosalicylate mesalazine (5-ASA), has been reported to act by inhibiting the Wnt/β-catenin pathway activity [23], and 5-ASA has been suggested as a chemopreventive in sporadic colorectal neoplasia via ß-catenin signaling [24].

In this study we analyzed, by immunohistochemistry, the expression of some crucial molecules involved in the signal transmission initiated by Wnt ligands, in colorectal inflammatory disease and during the CRC carcinogenetic process. Specifically, we analyzed upstream events of Wnt signaling including β-catenin nuclear translocation and loss of E-cadherin and APC functions, and downstream events including c-Myc and Cyclin-D1 nuclear expression. We performed a comparative evaluation of these molecules in an animal model of chemically-induced CRC and in human tissues obtained from patients suffering from IBD or at sequential stages of SCC, from low grade dysplasia to advanced carcinoma. The animal model used in this study consisted in syngenic immunocompetent BDIX rats, set up in our laboratory, in which colorectal adenocarcinoma was chemically induced by 1,2-dimethylhydrazine (DMH) administration. The DMH rodent models are widely used to study chemically-induced colon cancer [25, 26], and provide a useful preclinical model for studying early carcinogenesis and sporadic cancer development. The main purposes of the study were: i) To validate the animal model as a preclinical model of colorectal cancer that mimics the molecular events of Wnt signaling during IBD and SCC carcinogenesis in humans and that may therefore be useful for testing chemopreventive or therapeutic drugs targeting this pathway; ii) to assess the diagnostic and predictive values, in terms of responsiveness to Wnt-targeting therapies, of the Wnt/β-catenin signaling components, using a multiparametric approach.

## RESULTS

### Histological validation of the DMH rat model

**Figure 1:**
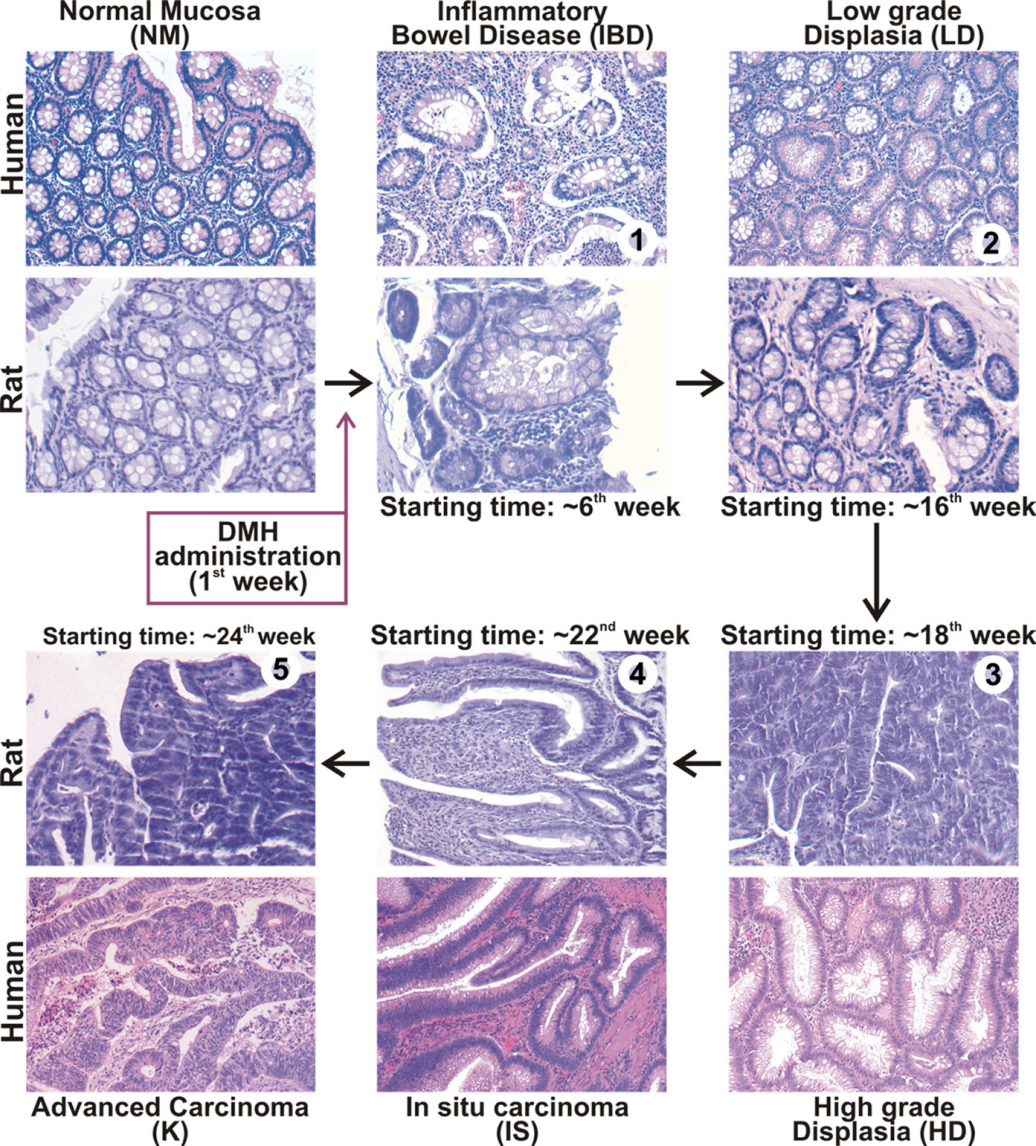
Histology of colonic mucosa in rat model of DMH-induced colorectal cancer Resuming scheme reporting the histology of normal mucosa (NM), IBD, dysplasia and colorectal cancer and the timing of the sequential steps during DMH-induced colon carcinogenesis in BDIX rats, compared with human tissues. Sections from rat colon resected from the 6^th^ to the 30^th^ week after the first DMH administration were subjected to histological examination, and compared to human specimens diagnosed as IBD, low grade dysplasia (LD), high grade dysplasia (HD), *in situ* carcinoma (IS) or advanced carcinoma (K). Colon from untreated animals or normal colon biopsy specimens were used as reference for normal mucosa (NM) morphology.

We preliminary assessed whether our experimental model of DMH-induced colon carcinogenesis in BDIX rats mimicked the histopatological lesion of IBD, dysplasia and sporadic colorectal cancer recovered in humans, and defined the timing of the sequential steps during cancer insurgence and progression in rats. Results are resumed in Figure 1. Starting from the 6^th^ week after the first DMH-administration, IBD-like morphological modifications were recovered, with crypt architectural distortion, inflammatory infiltrations in the *lamina propria* and basal plasmacytosis (Figure 1, step1). The areas of colonic mucosa exhibiting these IBD-like alterations became more represented with the on-going of time, up to the 14^th^ week. Starting from the 16^th^ week, rat tissue showed histological features of dysplastic colorectal epithelium, with hyperchromatic, crowded and elongated nuclei confined to the basal half of the epithelial cells, and sparse goblet mucinous cells, similarly to the LD recovered in human tissues (Figure 1, step2). Starting from the 18^th^ weeks, histological features of HD were prominent, with colon epithelial cells showing stratified, enlarged, pleomorphic and hyperchromatic nuclei, with cytoplasmic mucin vacuoles inconspicuous and almost absent (Figure 1, step3). Colorectal tissues explanted from rats after 22-24 weeks from the first DMH-administration, showed histopathological characteristics of well differentiated and moderately differentiated adenocarcinoma, with glandular structures present in more than 50% of total tissue, and submucosal invasion confined to *lamina propria*, similarly to human IS (Figure 1, step4 ). Starting from the 24^th^ week, IS areas were mixed with areas exhibiting histological features of K, with poorly differentiated and undifferentiated morphology, with rare glandular structures (less than 50% of total tissue) and submucosal invasion through the *muscularis* mucosa (Figure 1, step5).

### Comparative analysis in humans and rats of Wnt/β-catenin components

**Figure 2:**
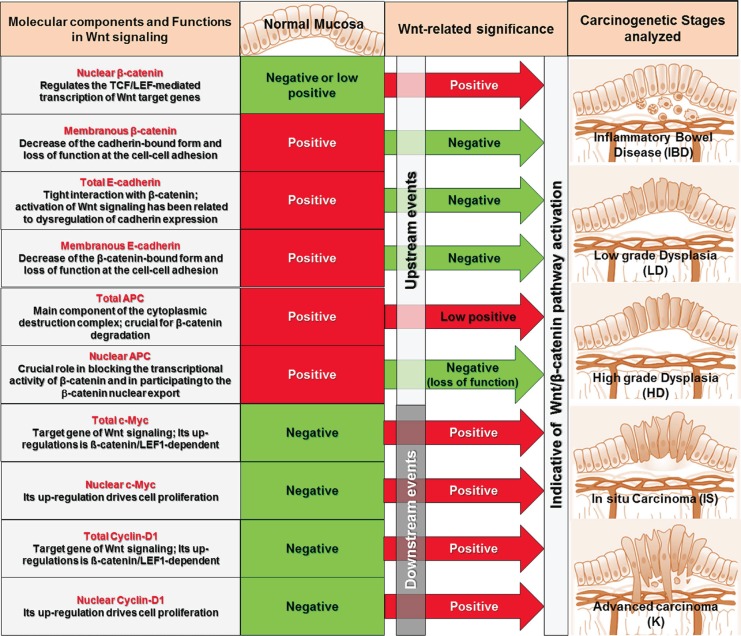
Resuming scheme of the Wnt pathway components and carcinogenetic stages analyzed in this study For each molecular components, the function in Wnt signaling (left column), the expression pattern in normal mucosa in absence of Wnt signaling (second column) and the modification indicative of Wnt signaling activation (third column), are reported.

Colorectal tissues explanted from rats and classified in the different histopathological groups (IBD, LD, HD, IS and K) were compared with human tissues, obtained from patients suffering from IBD and at the sequential stages of sporadic colorectal cancer, for the expression of the Wnt pathway components reported in the scheme of Figure 2. Specifically we analyzed the onco-protein ß-catenin, the epithelial differentiation marker E-cadherin, the tumor suppressor APC, and the two onco-proteins c-Myc and Cyclin-D1, which up-regulations are ß-catenin/LEF1-dependent and drive cell proliferation. Since the subcellular localization of a protein can provide important clues on its function (Figure 2), for each molecules we performed a quantitative analysis not only of the expression (total) but also of the intracellular distribution (nuclear and/or membranous), following the criteria reported in Materials and Methods and Table 1. Colorectal tissues from untreated rats or healthy patients were used as reference of the expression and distribution of the molecules examined in normal mucosa. For the quantitative analyses, mean score values and significance (*P* by the Student's *t* test) for each carcinogenetic stage *vs* NM and *vs* the previous stage are reported in Supplementary Table S2.

**Table 1:** Resuming table of the criteria used for quantifying immunohistochemical staining and arbitrary score values assigned

Biomarker expression	
Intensity vs CtrII Step	Score Value assigned
− /± (same as the background or few higher than the background)	1
+ (higher than the background)	10
++ (much higher than the background)	20
Biomarker subcellular localization	
% of positive cells	Score Value assigned
< or near 10%	5
>10% and <50%	10
>50%	20

### β-catenin during carcinogenesis

**Figure 3:**
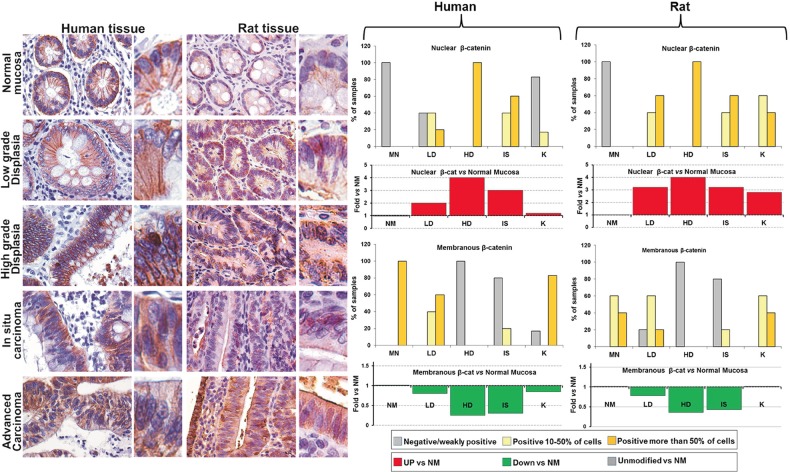
Comparative analysis of β-catenin expression and subcellular localization during the colorectal carcinogenesis in humans and rats Left panels: Immunohistochemical analysis of colorectal tissues explanted from rats and classified in the different histopathological groups (LD, HD, IS and K), compared with human tissues obtained from patients at the sequential stages of SCC from low grade dysplasia (LD) to invasive adenocarcinoma (K); for each representative image, a detail at higher magnification of colonic epithelial cells is shown on the right inset. Original magnification (OM): 20x, inset: 40x. Right panels: quantitative analyses of the expression and localization (nuclear and membranous), estimated as percentage of samples in which positive cells were less than 10% (negative or weakly positive), from 10 to 50%, and more than 50% of the cells constituting the epithelial mucosa (top bar graphs), and as trend *vs* normal mucosa (NM), reported as Fold vs NM, calculated as described in Materials and Methods (bottom bar graphs).

In all samples of normal mucosa from humans and rats, β-catenin staining localized at the cell membrane, while cytoplasmic and nuclear staining was generally absent (Figure 3). In human and rat LD, membranous β-catenin was still present in the majority of colonic epithelial cells but β-catenin cytoplasmic and nuclear staining was also observed in numerous cells. Specifically, 2 human LD tissues were negative for nuclear β-catenin while the remaining 3 showed nuclear positivity in 10-50% of cells (2 out of 3 samples) or in more than 50% of cells (1 out of 3 sample). In rat LD tissues, a quite similar result was obtained, with nuclear positivity observed in 10-50% of cells (2 out of 5 samples) or in more than 50% of cells (3 out of 5 samples). In all samples of HD from humans and rats, β-catenin staining localized mainly in the nucleus (more than 50% of colonic epithelial cells), and less in the cytoplasm, and membranous β-catenin was negative or weakly positive (less that 10% of positive cells). In human and rat IS tissues, nuclear β-catenin is still present in the majority of cells of all samples (Figure 3, upper bar graphs), but membranous β-catenin staining was also observed in 1 out of 5 samples, that exhibited 10-50% of positive cells. Finally, in advanced carcinoma from humans, membranous β-catenin was predominant *vs* nuclear β-catenin, that was observed only in 1 out of 5 samples, with 10-50% of positive nuclei. β-catenin localization at the cell membrane was also observed in the 100% of the rat tissues histologically classified as K, but, differently from humans, in rats all 5 samples also showed nuclear and cytoplasmic staining.

The trend of expression and distribution of β-catenin during the carcinogenentic process *vs* NM, quantified by assigning a score for each pattern of staining intensity and subcellular localization (Supplementary Table S2 and Supplementary Figure S1), was reported in Figure 3 as Fold *vs* NM. Despite the few differences observed in the advanced carcinoma, a similar trend of β-catenin expression was recorded in human and rat samples. Specifically, from LD to IS, nuclear β-catenin was up-regulated *vs* NM (Figure 3, red bars in the graphs) while membranous β-catenin was significantly down-regulated *vs* NM (Figure 3, green bars the in graphs), reaching the maxima values of up- and down-regulation, respectively, in HD lesions. In human K, β-catenin exhibited a pattern of intracellular distribution near to NM.

### E-cadherin during carcinogenesis

**Figure 4:**
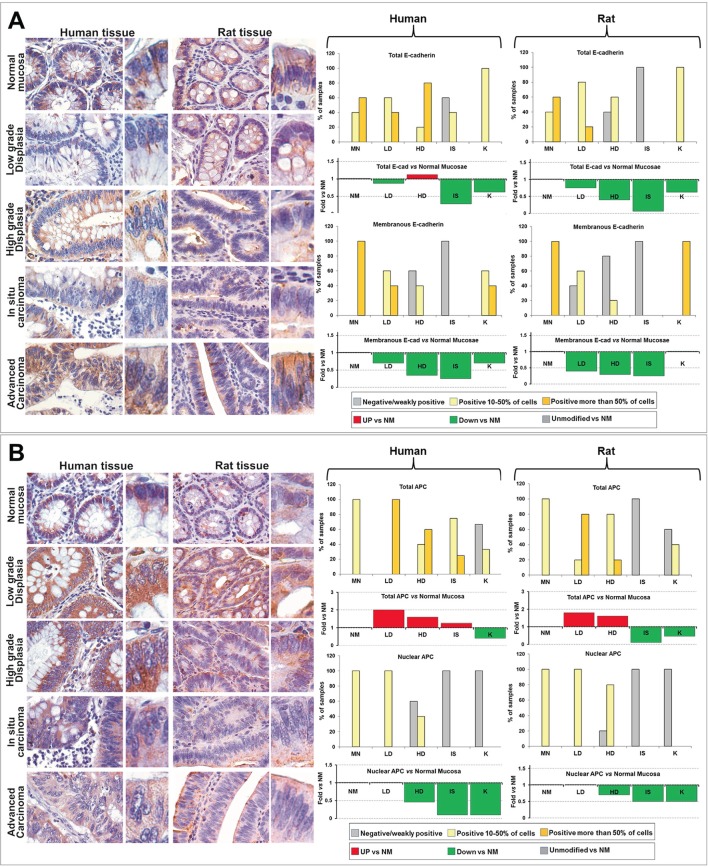
Comparative analysis of E-Cadherin (A) and APC (B) expression and subcellular localization during the colorectal carcinogenesis in humans and rats Left panels: Immunohistochemical analysis of colorectal rat tissues classified in the different histopathological groups (LD, HD, IS and K), compared with human tissues obtained from patients at the sequential stages of SCC from low grade dysplasia (LD) to invasive adenocarcinoma (K). OM: 20x, inset: 40x. Right panels: quantitative analyses of the expression and localization (total and nuclear), estimated as percentage of samples in which positive cells were less than 10% (negative or weakly positive), from 10 to 50%, and more than 50% of the cells constituting the epithelial mucosa (top bar graphs), and as trend *vs* normal mucosa (NM), reported as Fold *vs* NM (bottom bar graphs).

In normal mucosae from humans and rats, total E-cadherin was conspicuously expressed and localized almost exclusively at the cell membrane (Figure 4A). In human LD, membranous E-cadherin was still present in all samples analyzed but the number of positive cells was slightly lower than in NM (10-50% of positive cells in 3 out of 5 samples; more than 50% of positive cells in 2 out of 5 samples). In rat LD, E-cadherin was still expressed in the majority of colonic epithelial cells but 2 out of 3 samples showed less than 10% of positivity located on cell membrane (Figure 4A). Between HD and IS stages, E-cadherin expression was consistently reduced in both human and rat, and in IS lesions membranous E-cadherin was almost completely lost. Interestingly, in advanced carcinoma, 100% of human and rat samples showed E-cadherin expressed in 10-50% of colonic epithelial cells, mainly located at the cell-cell junctions (Figure 4A).

Although minor differences, in human and rat samples E-cadherin showed a similar trend of expression and localization from LD to K *vs* normal mucosa (Figure 4A and Supplementary Figure S1B). In particular, membranous E-cadherin, from LD to IS, was gradually down-regulated *vs* NM (Figure 4A, green bars in the graphs), reaching a minimum value between HD and IS lesions, while in K it tends to recover a pattern of expression similar to NM.

### APC during carcinogenesis

In all normal mucosae from humans and rats, total APC was expressed in the 10-50% of colonic epithelial cells and localized in the nucleus as well as in the cytoplasm (Figure 4B). In human and rat LD, the number of APC positive cells increased (more than 50% of positive cells), but the augmented expression was mainly observed in the cytoplasm, since the number of cells exhibiting nuclear positivity was unmodified as compared to NM. In HD lesions, only 2 out of 5 samples, from humans, and 4 out of 5 samples, from rats, exhibited APC in the nucleus (10-50% of positive nuclei), and in IS and K nuclear APC was almost completely negative or weakly positive in all samples examined.

As also reported for β-catenin and E-cadherin, in tissues from humans and rats, APC showed a similar trend of expression and localization from LD to K *vs* NM (Figure 4B and Supplementary Figure S1C). In particular, in LD, total APC was up-regulated *vs* NM (Figure 4B, red bars in the graphs), but, from LD to K, was gradually down-regulated, reaching minimum values between IS and K lesions. Concomitantly, from HD stage, APC nuclear localization decreased and at IS and K stages was almost completely negative.

### c-Myc and Cyclin-D1 during carcinogenesis

**Figure 5:**
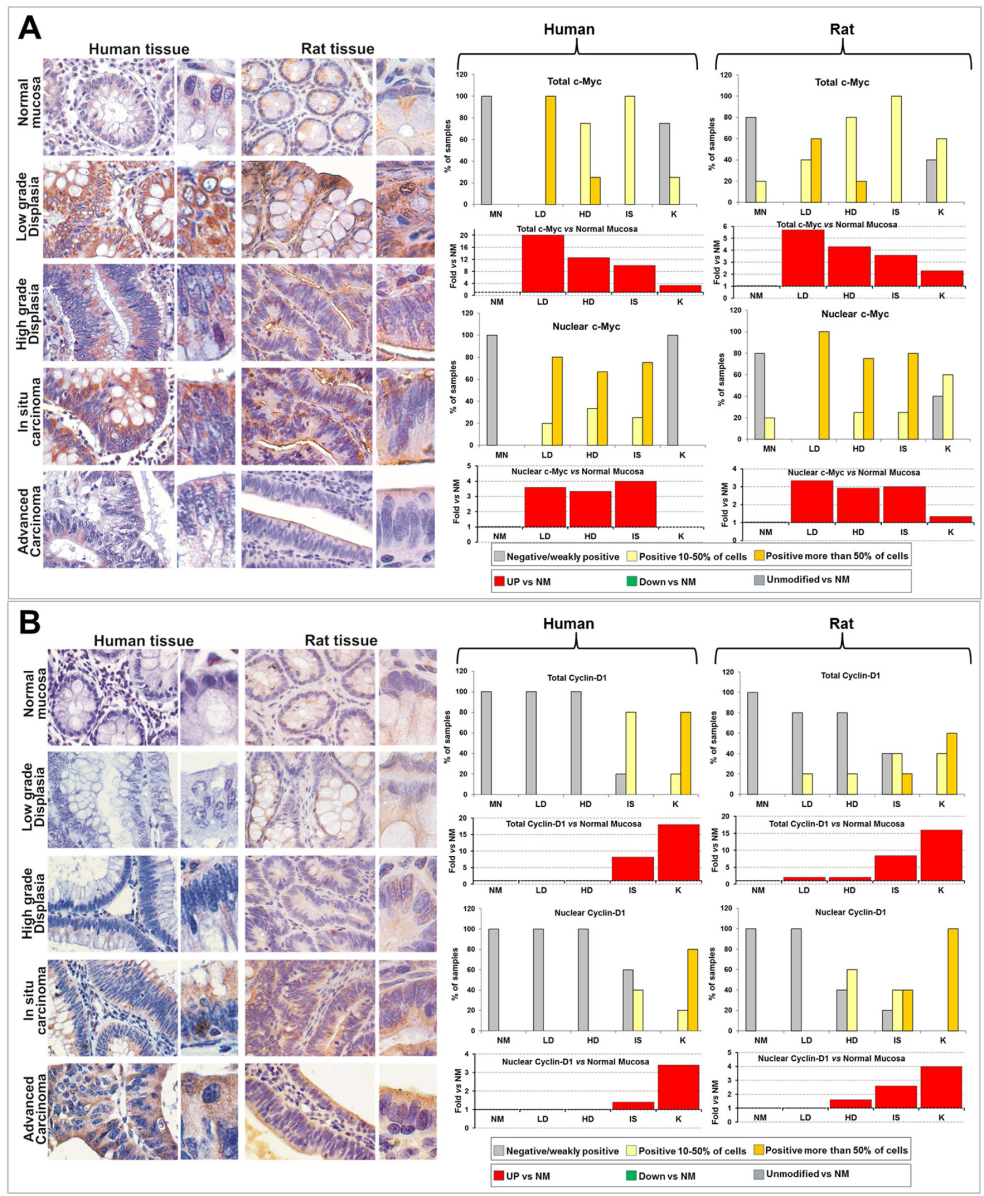
Comparative analysis of c-Myc (A) and Cyclin-D1 (B) expression and subcellular localization during the colorectal carcinogenesis in humans and rats Left panels: Immunohistochemical analysis of colorectal rat tissues classified in the different histopathological groups (LD, HD, IS and K), compared with human tissues obtained from patients at the sequential stages of SCC from low grade dysplasia (LD) to invasive adenocarcinoma (K). OM: 20x, inset: 40x. Right panels: quantitative analysis of the expression and localization (total and nuclear), estimated as percentage of samples in which positive cells were less than 10% (negative or weakly positive), from 10 to 50%, and more than 50% of the cells constituting the epithelial mucosa (top bar graphs), and as trend *vs* normal mucosa (NM), reported as Fold *vs* NM (bottom bar graphs).

In normal mucosae from humans and rats, total c-Myc and Cyclin-D1 were virtually absent (less than 10% of positive cells), with nuclei almost completely negative except for few c-Myc^+^ cells located at the basal portion of the crypts, where proliferative progenitors of colonic epithelial cells reside. In all samples of LD and HD from humans more that 50% of cells were c-Myc^+^ (Figure 5A), that localized mainly in the nucleus, while Cyclin-D1 was virtually not expressed (Figure 5B). In humans, nuclear positivity for c-Myc persisted in IS lesions, but 4 out of 5 samples also exhibited 10-50% of Cyclin-D1^+^ cells. In K, c-Myc was expressed only in 1 out of 5 samples, with nuclei virtually negative (Figure 5A), while Cyclin-D1 was expressed in all samples analyzed and exhibited nuclear localization in the majority of cells (Figure 5B).

Although minor differences, in human and rat samples c-Myc and Cyclin-D1 showed similar trends of expression and localization from LD to K *vs* NM (Figure 5 and Supplementary Figure S1D and E). In detail, c-Myc was dramatically up-regulated in LD *vs* NM (Figure 5A, red bars in the graphs), but, from LD to K, was gradually down-regulated even if it retained the nuclear localization, reaching a minimum value in K lesions. Concomitantly, Cyclin-D1, that was negative in low and high grade dysplastic lesions, was up-regulated in neoplastic lesions (IS and, even more, in K) *vs* NM, with a maximum value in advanced carcinoma (Figure 5B, red bars in the graphs).

### Expression of β-catenin, E-cadherin, APC, c-Myc and Cyclin-D1 in IBD

Owing the role recently ascribed to Wnt-pathway activation in IBD-associated carcinogenesis [22], we finally examined the expression and subcellular localization of β-catenin, E-cadherin, APC, c-Myc and Cyclin-D1, in rat colonic mucosae resected at the 6^th^-8^th^ weeks after the first DMH administration (histologically classified as IBD), and in human specimens from patients suffering from IBD (Chron's disease or ulcerative rectocolitis).

**Figure 6:**
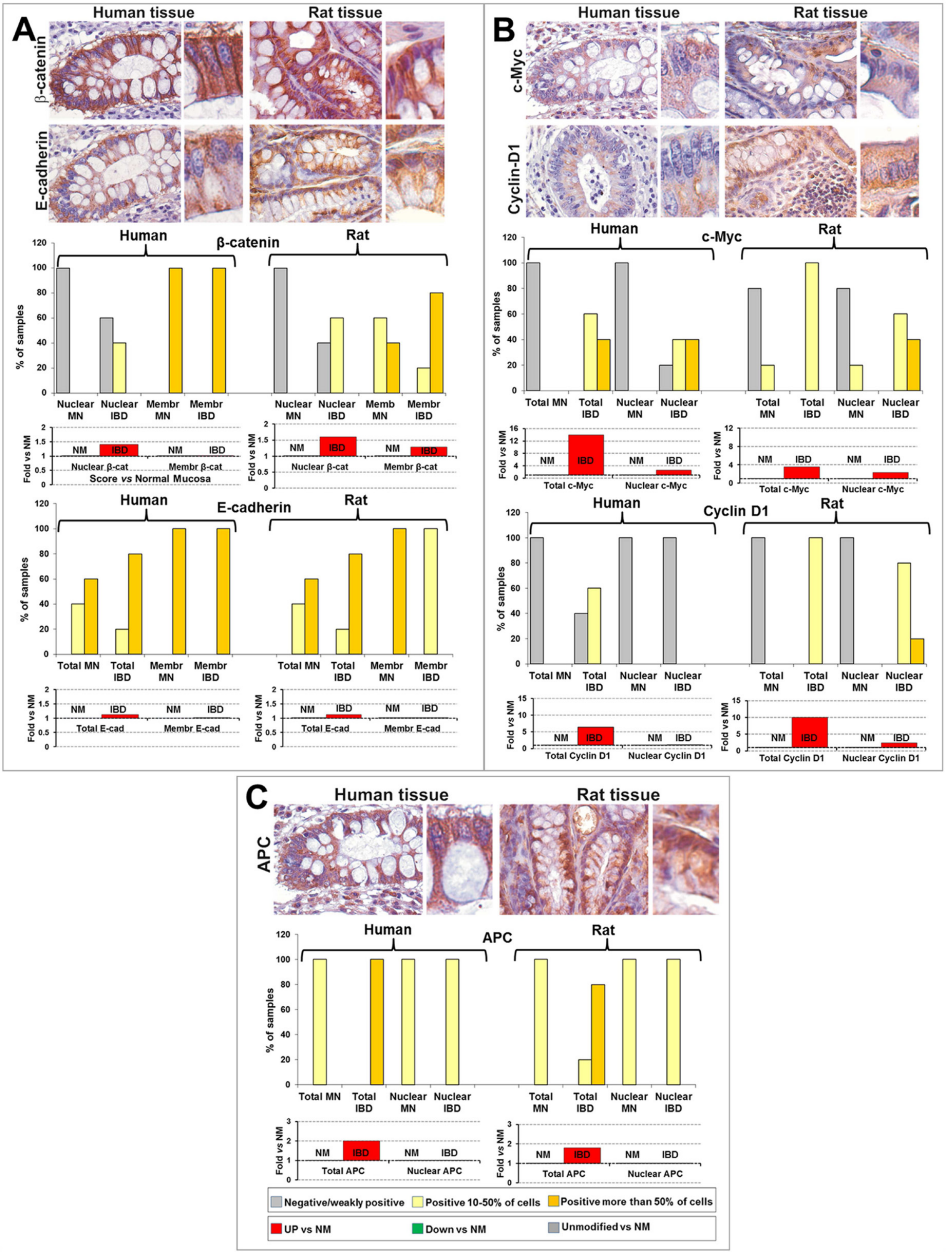
Comparative analysis of expression and subcellular localization of β-catenin and E-cadherin (A), c-Myc and Cyclin-D1 (B) and of APC (C) in IBD from humans and rats In each panel: representative images of the immunohistochemical analysis performed in human and rat IBD are shown; for each representative image, a detail at higher magnification of colonic epithelial cells is shown on the right inset; OM: 20x, inset: 40x. Bar graphs report the quantitative analyses of the expression and localization of each biomarker, estimated as percentage of samples in which positive cells were less than 10% (negative or weakly positive), from 10 to 50%, and more than 50% of the cells constituting the epithelial mucosa (top bar graphs), and as trend *vs* normal mucosa (NM), reported as Fold *vs* NM (bottom bar graphs).

In human and rat IBD tissues, membranous β-catenin was present in the majority of colonic epithelial cells, similarly to what observed in NM, while nuclear β-catenin was slightly up-regulated *vs* NM, with positive nuclei observed in 10-50% of colonic epithelial cells (Figure 6A and Supplementary Figure S1A). Conversely, E-cadherin expression and subcellular localization were virtually unmodified in IBD as compared to NM, even if in rat samples the percentage of cells exhibiting E-cadherin located at the cell membrane was lower than in NM (Figure 6A and Supplementary Figure S1B).

In IBD samples from humans and rats, APC exhibited nuclear localization in 10-50% of cells, as also observed in NM, but was up-regulated in total expression *vs* NM (2-fold and 1.8-fold in human and rat, respectively) (Figure 6C and Supplementary Figure S1C).

c-Myc expression and nuclear localization were significantly up-regulated in IBD *vs* NM from humans and rats (Figure 6B and Supplementary Figure S1D), even if with minor differences. Specifically, in human IBD total c-Myc was 14-fold higher *vs* NM, while in rats this increment was only of 3.6-fold. Conversely, in human IBD, Cyclin-D1 was not significantly modified *vs* NM (*P* ˃ 0.05, Supplementary Table S2), although of a weak increment of total expression was recorded, and it was virtually absent in cell nuclei (Figure 6B and Supplementary Figure S1E). Indeed, in rat IBD tissues Cyclin-D1 resulted dramatically up-regulated *vs* NM (10-fold for total expression), and in all samples analyzed this onco-protein localized in the nucleus of 10-50% of colonic epithelial cells (4 out of 5 samples) or in more than 50% of cells (1 sample) (Figure 6B).

### Multiparametric analysis of Wnt signaling components in IBD and during carcinogenesis

**Figure 7:**
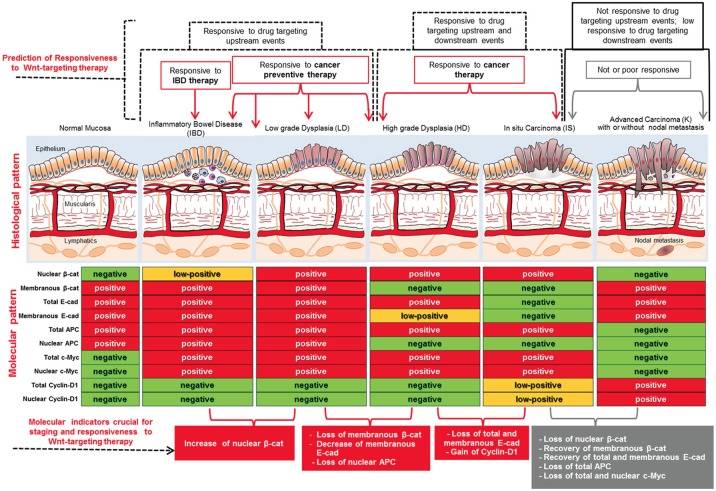
Resuming scheme of the multiparametric analysis performed on human tissues from IBD to K stages We combined the quantitative results (Supplementary Table S2) in a multiparametric panel including both expression and subcellular localization of all markers analyzed. negative: biomarkers with mean score values ≤ 6; positive: biomarkers with mean score values ≥10; low-positive: biomarkers with mean score values ˃6 and ˂10. Molecular changes that could be considered crucial for staging and responsiveness to Wnt-targeting drugs are indicated on the bottom. Hypothetical prediction of responsiveness to Wnt-targeting therapies is indicated on the top.

We finally combined the quantitative results obtained on human samples from IBD to K stages (Supplementary Table S2 and Supplementary Figure S1), in a multiparametric panel including both expression and subcellular localization of all markers analyzed (Figure 7). Using the criteria reported in Materials and Methods, we defined as negative the biomarkers with mean score values ≤ 6, positive those with mean score values ≥10 and low-positive the biomarkers that had mean scores from ˃6 and ˂10. As resumed in Figure 7, using this multiparametric approach each histopathological group (IBD, LD, HD, IS and K) resulted associated with a distinctive molecular pattern of expression and subcellular localization of β-catenin, E-cadherin, APC, c-Myc and Cyclin-D1. In general, upstream events of Wnt signaling, such as β-catenin nuclear translocation, were already present in IBD and at the early stage of LD, but in LD nuclear β-catenin increased from low-positive to positive as compared to IBD. Going ahead, loss of nuclear APC characterized the passage from LD to HD while loss of E-cadherin occurred between HD and IS.

As it concerns events downstream the ß-catenin/LEF1 interaction, nuclear c-Myc expression was positive already in IBD and at the early stages of neoplastic transformation and was lost between IS and K, while Cyclin-D1 expression characterized the later stages of *in situ* and, even more, advanced carcinoma. Upstream and downstream events of Wnt signaling (Figure 2) seemed to be wholly activated in the IS stage, while at K stage the molecular pattern resembled to normal mucosa except for the high positivity for Cyclin-D1 and the absence of nuclear APC.

## DISCUSSION

Nowadays, each existing biomarker used or proposed for CRC early diagnosis, staging and prognosis alone is poorly specific and the absolute positive and negative serological and/or immunohistochemical markers are still lacking. The more promising approach for developing more specific diagnostic/prognostic tools might be to combine several positive or negative indicators in multiparametric platforms, that allow simultaneous detection of multiple serological or immunohistochemical markers for CRC. Moreover, since target-specific cancer therapy has remarkably improved the outcomes of patients and represents the frontline approach for cancer treatment, the development of such multiparametric platforms would also represent high-performance technological tools useful for designing personalized therapies, adapted to the aggressiveness of each individual tumor.

In the last years the researches on the molecular mechanisms driving the Wnt/β-catenin signaling pathway are in increasing and conspicuous funds are invested by several pharmaceutical and biotech companies for the development of innovative antitumor drugs targeting its molecular components [26]. Indeed, Wnt/β-catenin pathway activation not only is involved in colorectal cancer development but also seems to be a common event to IBD and early stages of colon carcinogenesis [22], and deregulation or constitutive activation of this pathway could represent the connection between colonic chronic inflammation and increased risk of developing SCC. Thus, drugs targeting this pathway could be also useful for developing chemopreventive strategies against CRC [17, 19, 20, 27, 28].

In this context, knowledge on the sequential molecular events of Wnt-signaling cascade in IBD and during colon carcinogenesis, in addition to providing new diagnostic/prognostic markers, could be helpful for optimizing the treatment protocol and improve the therapeutic efficacy of these drugs.

In this study, we focused our analyses on ß-catenin, E-cadherin, APC, c-Myc and Cyclin-D1, which expressions and subcellular localizations could be indicative of Wnt signaling activation. Specifically, the first molecule explored was the onco-protein ß-catenin, which stabilization and nuclear translocation regulate the TCF/LEF-mediated transcription of Wnt pathway target genes [16-20]. Thus, ß-catenin decrease at the cell membrane and its nuclear import have been used as main indicators of Wnt pathway activation. The second molecules analyzed was the epithelial differentiation marker E-cadherin. Due to the tight interaction of β-catenin with E-cadherin at the cell-cell junction, activation of Wnt signaling has also been related to dysregulation of cadherin expression, which is often associated with dysplasia, tumor formation, and metastasis [29, 30]. This causal relationship between E-cadherin and Wnt signaling makes E-cadherin an additional molecular marker that we took into account in the setting up of our analyses. The third molecule that we analyzed was the onco-suppressor APC that, besides its function at the destruction complex, in the nucleus has a crucial role in blocking the transcriptional activity of β-catenin and in participating to the β-catenin nuclear export toward the cytoplasm either for degradation or for assembly at adherent junctions [31, 32]. For this reason, in our analyses APC nuclear localization was considered as an indicator of APC functionality and was evaluated together with total APC expression. Finally, total expression and nuclear localization of the onco-proteins c-Myc and Cyclin-D1, which up-regulations are ß-catenin/LEF1-dependent, have been analyzed as additional biomarkers indicative of downstream events of Wnt signaling.

The first result that we obtained in this study was the validation of our pre-clinical model of CRC. As in other DMH-induced rodent model, in our rat model of colorectal cancer, carcinogenesis develops through a multistep process similar to human colon carcinogenesis (Figure 1). In this pre-clinical model we determined the timing of the sequential steps of the carcinogenetic process, from IBD (6^th^ week after the first DMH administration) to LD, HD, IS and K (from 16^th^, 18^th^, 22^nd^ and 24^th^ week, respectively). Moreover, the comparative evaluation of ß-catenin, E-cadherin, APC, c-Myc and Cyclin-D1 in rat and in human, demonstrated that our preclinical model, although minor differences, mimics the molecular events of Wnt signaling during IBD and SCC carcinogenesis in humans. Indeed, we recorded, already in IBD and in early stages of neoplastic transformation (LD and HD), changes in the expression and tissue distribution of these molecules, indicative of Wnt/ß-catenin pathway activation, while Cyclin-D1 was modified at later stages, in IS and, even more, in K. Indeed, the Wnt/ß-catenin pathway have an active role from IBD to IS, with upstream and downstream events of Wnt signaling wholly activated at the IS stage, while in advanced carcinoma the role of this pathway seems to overshadow, as indicated by the absence on nuclear ß-catenin.

The most important and innovative result concerns the design of a multiparametric panel obtained by combining both expression and subcellular localization of all molecules analyzed (Figure 7). By assigning an arbitrary score to expression level and localization of each biomarker examined, and defining two cut-off for classifying negative, positive and low-positive results, we propose an algorithm that could be useful to the histopathologists and oncologists for diagnostic and therapeutic purposes (Figure 7). We obtained evidence that this multiparametric analysis, when combined with the histology, not only might allow a more accurate diagnosis, by discriminating between early and late phase within each stage, but could also predict response to Wnt-targeting chemotherapy (Figure 7). The main therapeutic approaches that are currently explored in this field, some of which are under Phase 1/2 clinical investigation (Supplementary Table S3) [33], imply the use of drugs targeting molecules participating to upstream events, such as Wnt ligands [34], Fzd receptors [35], LPR co-receptors [36, 37], Dishevelled [38] and others [27], or drugs targeting downstream events such as β-catenin/TCF or β-catenin/CBP interactions [39, 40]. As schematized in Figure 7, our multiparametric approach suggests that: i) drugs targeting upstream events could be useful for chemopreventive strategies and for treatment of patients suffering for IBD, low and high dysplasia as well as non-invasive carcinoma; ii) drugs targeting downstream events could be more effective when used for treatment of late HD and IS; iii) the advanced carcinoma could be not responsive to drugs targeting upstream events and poor responsive to drugs targeting downstream events.

In conclusion, we demonstrated that crucial components of the Wnt/β-catenin pathway, when evaluated by immunohistochemistry using a multiparametric approach that includes the analysis of both expression and localization, could be potent markers for diagnosis, prevention and therapy in IBD and sporadic colorectal cancer, also possessing a predictive value for responsiveness to Wnt-targeting therapies. Even if the biomarkers included in this study should be validated with a larger number of samples to increase the statistical significance, our results provide the basis for the development of a multiparametric platform that might be used to design “specific maps” for CRC early diagnosis and staging, and that would represent an useful tool for determining the responsiveness to new drugs targeting the Wnt signaling. Even if clinically useful agents that specifically inhibit Wnt signaling cascade are not currently available, several therapeutic strategies in this sense are in currently developing and need to be validate [27, 34-39]. In this context, the model of DMH-induced carcinogenesis in rats, that mimics the molecular events of Wnt signaling during IBD and SCC carcinogenesis in humans, may be a useful preclinical model for evaluating the chemopreventive or therapeutic efficacy of these drugs.

## MATERIALS AND METHODS

### Animal model of colorectal carcinogenesis and animal tissue specimens

Inbred male BDIX rats (Charles River, Calco, Italy), 8 weeks old and weighing 220–250g, were housed for 7 days, in a pathogen-free animal facility with free access to water and food in accordance with European Community guidelines. Experiments were approved by the local committee on animal experimentation, and were performed under strict governmental and international guidelines. Following a 1-week acclimation period, and after rat anesthetization by inhalation of 1-bromo-2-chloro-1,1,1-trifluoroethane (Fluka, Sigma-Aldrich, St. Louis, MO), carcinogenesis was induced by DMH administered subcutaneously at the dose of 30mg/kg, once a week for 5 weeks. Untreated animals (*n*=5) were used as control group to obtain normal colonic mucosa. DMH-treated rats (*n*=5 animals for each time point) were sacrificed, by inhalation of an overdose of CO_2_, once every two week, starting from the 6^th^ to the 30^th^ week after the first DMH administration. At autopsy, the entire colon rectum was excised, flushed with saline, pre-fixed in 10% buffered formalin for 2h and cut transversally in ring-shaped pieces of about 1mm thick. After additional 22h of formalin fixation, samples were embedded in paraffin in a routine manner for histological and immunohistochemical analyses. Sections from each paraffin block of rat or human tissues were stained with H&E for the histological examination.

### Human specimens

Paraffin-embedded human colon specimens (*n*=30), taken for diagnostic purposes or after surgical excision of localized disease, were collected from pathology archives and analyzed anonymously in adherence to ethical standards. In this study we used the following samples: 5 specimens from patients suffering from IBD (Chron's disease or ulcerative rectocolitis), 5 from low grade dysplasia (LD), 5 from high grade dysplasia (HD), 5 from *in situ* carcinoma (IS) and 5 from advanced carcinoma (K). Normal colon biopsy specimens (NM, *n*=5) were obtained from patients in whom colonoscopy was performed to rule out colorectal disease. The diagnosis was based on histopathological examination of routinely processed tissue together with clinical and laboratory data. Serial sections from each sample were subjected to hematoxylin-eosin (H&E) staining or to immunohistochemical analyses as described below.

### Histology, immunohistochemical analyses and scoring

Sections from each paraffin block of rat or human tissues were stained with H&E for the histological examination. Based on histological features, each rat sample, obtained from the control group and from the DMH-treated group, was classified as normal mucosa (NM) or included within the different histopathological groups (IBD, LD, HD, IS and K). For immunohistochemical analyses, serial sections from rat or human tissues were collected on SuperFrost® Plus coated slides (Menzel-gläser, Braunschweig, Germany) and immuno-stained for ß-catenin, E-cadherin, APC, c-Myc and Cyclin-D1, using the specific antibodies and the working dilution reported in Supplementary Table S1. The specificity of each antibody has been preliminary assessed by Western blot analysis, taking into account the production of a single band matched to the proper molecular weight, as previously reported [41]. Tissue sections were incubated in 1% BSA for 15min at room temperature to block non-specific background, and then incubated overnight with the specific antibodies. Primary antibody reaction was revealed using the KIT DAKO Cytomation LSAB 2® System HRP (Liquid DAB) that employs the streptavidin-biotin complex method. After peroxidase reaction, sections were counterstained with hematoxylin. For each specimen examined and on a section close to that used for immuno-staining, background controls were performed by omitting the specific primary antibody (Ctr II step). In all samples from patients and rats, the quantitative analyses of the expression (total) and distribution (nuclear and/or membranous) of all molecules analyzed was performed by evaluating, for each histopathological group as well as in normal mucosa:

the percentage of samples in which positive cells were less than 10% (negative or weakly positive), from 10 to 50%, and more than 50% of the cells constituting the epithelial mucosa;

the staining intensity (for total expression) and the percentage of positive cells (for nuclear or membranous localization), as compared to the background control (Ctr II step), following the criteria reported in Table 1. For each staining pattern of expression and localization, a score value was arbitrary assigned (Table 1) and the mean scores, obtained for each marker from all samples classified in the same histopathological group (HpG), were plotted. The trend of expression and intracellular distribution of the different markers analyzed *vs* normal mucosa (NM) has been also reported as fold *vs* NM, calculated by the formula:

$$\text{Fold }vs\text{ NM}=\frac{\text{Mean Score in HpG}}{\text{Mean Score in NM}}$$

For the multiparametric analysis of all molecules analyzed, we defined the following cut-off for negative, positive and low-positive results, based on the arbitrary score assigned for each staining pattern of expression and localization (Table 1):

negative: biomarkers with mean score values ≤ 6, i.e. the sum of score assigned to −/± intensity (score=1) plus score assigned to % of positive cells ˂10% (score=5)

positive: biomarkers with mean score values ≥10

low-positive: biomarkers with mean score values ˃6 and ˂10

### Statistical analysis

For statistical analysis, groups were compared using the two-tailed Student's *t* test and a *P* value threshold of < 0.05. Data of the mean scores obtained for each biomarker were presented as mean ± SD. (*P < 0.05; **P < 0.01; ***P < 0.001)

## SUPPLEMENTARY FIGURE AND TABLES




