# Supplementary material for: WNT-pathway components as predictive markers useful for diagnosis, prevention and therapy in inflammatory bowel disease and sporadic colorectal cancer
Source: Oncotarget. 2014 Jan 11;5(4):978–92. doi: 10.18632/oncotarget.1571 (PMC4011599; doi:10.18632/oncotarget.1571)
Supplement: Supplementary file 1 [file oncotarget-05-978-s001.pdf]

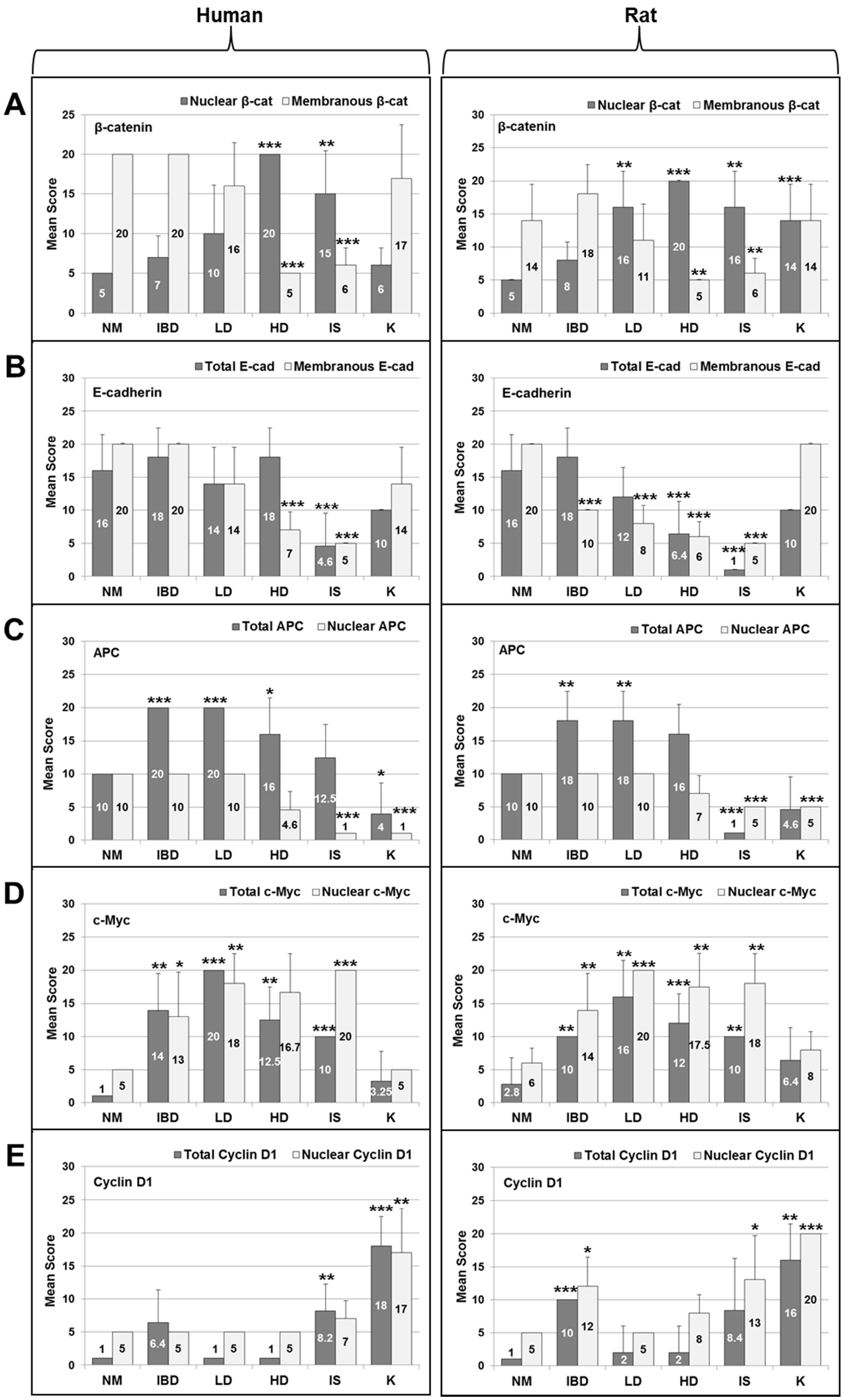
Human
Rat
A
B
C
D
E
Nuclear β-cat
Membranous β-cat
β-catenin
Total E-cad
Membranous E-cad
E-cadherin
Total APC
Nuclear APC
APC
Total c-Myc
Nuclear c-Myc
c-Myc
Total Cyclin D1
Nuclear Cyclin D1
Cyclin D1
Mean Score
NM
IBD
LD
HD
IS
K

Supplementary Figure S1. Results of the quantitative analyses of all marker analyzed in humans and rats, from IBD to K stages. For each biomarker, the quantitative analysis was performed evaluating the staining intensity (for total expression) and the percentage of positive cells (for nuclear or membranous localization), as compared to the background control (Ctr II step), following the criteria reported in Table 1. For each staining pattern of expression and localization, a score value was arbitrary assigned (Table 1) and the mean scores, obtained for each marker from all samples classified in the same histopathological group were plotted. For statistical analysis, groups were compared using the two-tailed Student's *t* test and a *P* value threshold of < 0.05. Data of the mean scores obtained for each biomarker were presented as mean ± SD. Significance vs NM: (**P* < 0.05; ***P* < 0.01; ****P* < 0.001). The mean score values plotted and significance for each carcinogenetic stage *vs* NM and *vs* the previous stage are reported in Supplementary Table S2.
