# Supplementary material for: WNT-pathway components as predictive markers useful for diagnosis, prevention and therapy in inflammatory bowel disease and sporadic colorectal cancer
Source: Oncotarget. 2014 Jan 11;5(4):978–92. doi: 10.18632/oncotarget.1571 (PMC4011599; doi:10.18632/oncotarget.1571)
Supplement: Supplementary file 2 [file oncotarget-05-978-s002.pdf]

Supplementary Table S1. List of antibodies used for immunohistochemical analysis

Antigen	Host	Cat. #	Working dilution	Supplier
β-catenin	Mouse (monoclonal)	610154 (Clone 14)	1:150	BD Transduction Labs (Palo Alto, CA, USA)
E-cadherin	Mouse (monoclonal)	610182 (Clone 36)	1:200	BD Transduction Labs (Palo Alto, CA, USA)
APC	Rabbit (monoclonal)	ab40778	1:100	Abcam (Cambridge, MA, USA)
c-Myc	Mouse (monoclonal)	sc-40	1:50	Santa Cruz Biotechnology (Dallas, TX, USA)
Cyclin-D1	Mouse (monoclonal)	sc-8396	1:50	Santa Cruz Biotechnology (Dallas, TX, USA)

Supplementary Table S2. Mean score values and significance (Student's *t* Test) of the quantitative analysis reported in Supplementary Figure S1. *P* value threshold: *P* < 0.05. Significant values are highlighted in red

HUMAN	Mean score	±S.D.	*P vs* NM	*P vs* previous Stage		RAT	Mean score	±S.D.	*P vs* NM	*P vs* previous Stage	
Nuclear β-catenin											
**NM**	5	0.00				**NM**	5	0.00			
**IBD**	7	2.74	0.177807808			**IBD**	8	2.74	0.070483997		
**LD**	10	6.12	0.141927448	0.20800000	*vs* IBD	**LD**	16	5.48	**0.010899699**	**0.002837846**	*vs* IBD
**HD**	20	0.00	**1.18835x10^{-12}**	**0.022048174**	*vs* LD	**HD**	20	0.00	**1.18835x10^{-12}**	0.179236763	*vs* LD
**IS**	15	5.48	**0.010899699**	0.179236763	*vs* HD	**IS**	16	5.48	**0.010899699**	0.179236763	*vs* HD
**K**	6	2.24	0.373900966	**0.011056493**	*vs* IS	**K**	14	5.48	**0.021311641**	0.373900966	*vs* IS
Membranous β-catenin											
**NM**	20	0.00				**NM**	14	5.48			
**IBD**	20	0.00	0.373900966			**IBD**	18	4.47	0.177807808		
**LD**	16	5.48	0.177807808	0.179236763	*vs* IBD	**LD**	11	5.48	0.208000000	**0.024896163**	*vs* IBD
**HD**	5	0.02	**1.18203x10^{-12}**	**0.010928395**	*vs* LD	**HD**	5	0.00	**0.021311641**	0.070483997	*vs* LD
**IS**	6	2.24	**0.000151011**	0.373900966	*vs* HD	**IS**	6	2.24	**0.01613009**	0.373900966	*vs* HD
**K**	17	6.71	0.373900966	**0.051374431**	*vs* IS	**K**	14	5.34	0.373900966	**0.01619462**	*vs* IS
Total E-cadherin											
**NM**	16	5.48				**NM**	16	5.48			
**IBD**	18	4.47	0.373900966			**IBD**	18	4.47	0.373900966		
**LD**	14	5.48	0.373900966	0.177807808	*vs* IBD	**LD**	12	4.47	0.177807808	0.070483997	*vs* IBD
**HD**	18	4.47	0.373900966	0.177807808	*vs* LD	**HD**	6,4	4.93	**2.53213x10^{-6}**	0.071076798	*vs* LD
**IS**	4,6	4.93	**0.003981595**	**0.004278734**	*vs* HD	**IS**	1	0.00	**0.003602233**	0.070483997	*vs* HD
**K**	10	0.00	0.070483997	0.070483997	*vs* IS	**K**	10	0.00	0.070489127	**1.4762x10^{-10}**	*vs* IS
Membranous E-cadherin											
**NM**	20	0.00				**NM**	20	0.00			
**IBD**	20	0.00	0.373900966			**IBD**	10	0.00	**9.5233x10^{-11}**		
**LD**	14	5.48	0.070483997	0.071262472	*vs* IBD	**LD**	8	2.74	**0.000608185**	0.183594823	*vs* IBD
**HD**	7	2.74	**0.000445954**	**0.004635839**	*vs* LD	**HD**	6	2.24	**0.000151011**	0.177807808	*vs* LD
**IS**	5	0.00	**1.88619x10^{-11}**	0.177835707	*vs* HD	**IS**	5	0.00	**1.8862x10^{-11}**	0.373900966	*vs* HD
**K**	14	5.48	0.070483997	**0.021504336**	*vs* IS	**K**	20	0.00	0.373900966	**1.2382x10^{-11}**	*vs* IS

HUMAN	Mean score	±S.D.	*P vs* NM	*P vs* previous Stage		RAT	Mean score	±S.D.	*P vs* NM	*P vs* previous Stage	
**Total APC**											
**NM**	10	0.00				**NM**	10	0.00			
**IBD**	20	0.00	**4.32343×10^{-9}**			**IBD**	18	4.47	**0.01613009**		
**LD**	20	0.00	**2.76078×10^{-10}**	0.649988954	*vs* IBD	**LD**	18	4.47	**0.01613009**	**0.01613009**	*vs* IBD
**HD**	16	5.48	0.057668886	0.178381187	*vs* LD	**HD**	16	4.47	0.373900966	0.070483997	*vs* LD
**IS**	12,5	5.00	0.391002219	0.181690114	*vs* HD	**IS**	1	0.00	**1.4645×10^{-14}**	**0.005336446**	*vs* HD
**K**	4	4.93	0.057668886	**4.34235×10^{-5}**	*vs* IS	**K**	4,6	4.93	0.070483997	0.178124329	*vs* IS
**Nuclear APC**											
**NM**	10	0.00				**NM**	10	0.00			
**IBD**	10	0.00	0.422649731			**IBD**	10	0.00	0.422649731		
**LD**	10	0.00	0.422649731	0.547089186	*vs* IBD	**LD**	10	0.00	0.422649731	1	*vs* IBD
**HD**	4,6	2.74	0.422649731	0.072048725	*vs* LD	**HD**	7	2.74	0.422649731	0.070484204	*vs* LD
**IS**	1	0.00	**4.38548×10^{-5}**	0.177835707	*vs* HD	**IS**	5	0.00	**4.43852×10^{-7}**	0.177808092	*vs* HD
**K**	1	0.00	**4.43852×10^{-7}**	0.43137677	*vs* IS	**K**	5	0.00	**4.43852×10^{-7}**	1	*vs* IS
**Total c-Myc**											
**NM**	1	0.00				**NM**	2,8	4.02			
**IBD**	14	5.48	**0.006057526**			**IBD**	10	0.00	**0.01613009**		
**LD**	20	0.00	**4.61371×10^{-13}**	0.071262472	*vs* IBD	**LD**	16	5.48	**0.005129326**	0.070483997	*vs* IBD
**HD**	12,5	5.00	0.019312445	**0.057668886**	*vs* LD	**HD**	12	4.47	**1.33584×10^{-6}**	0.177807808	*vs* LD
**IS**	10	0.00	**1.37276×10^{-7}**	0.422649731	*vs* HD	**IS**	10	0.00	**0.01613009**	0.373900966	*vs* HD
**K**	3,25	4.50	0.391002219	**1.37276E-07**	*vs* IS	**K**	6,4	4.93	0.177807808	0.177807808	*vs* IS
**Nuclear c-Myc**											
**NM**	5	0.00				**NM**	6	2.24			
**IBD**	13	6.71	**0.056**			**IBD**	14	5.48	**0.01613009**		
**LD**	18	4.47	**0.002888981**	0.088987311	*vs* IBD	**LD**	20	0.00	**0.000151011**	0.070483997	*vs* IBD
**HD**	16,67	5.77	0.07282735	0.422649731	*vs* LD	**HD**	17,5	5.00	**0.015392438**	0.391002219	*vs* LD
**IS**	20	0.00	**4.94047×10^{-8}**	0.423227369	*vs* HD	**IS**	18	4.47	**0.01613009**	0.391002219	*vs* HD
**K**	5	0.00	0.373900966	**4.94047E-08**	*vs* IS	**K**	8	2.74	0.177807808	**0.021742978**	*vs* IS
**Total Cyclin-D1**											
**NM**	1	0.00				**NM**	1	0.00			
**IBD**	6,4	4.93	0.070483997			**IBD**	10	0.00	**1.37276×10^{-7}**		
**LD**	1	0.00	0.373900966	0.070484061	*vs* IBD	**LD**	2	4.02	0.373900966	**1.37276×10^{-7}**	*vs* IBD

**HD**	1	0.00	0.373900966	0.43137677	*vs* LD	**HD**	2	4.02	0.373900966	0.373900966	*vs* LD
**IS**	8,2	4.02	**0.01613009**	**0.01613009**	*vs* HD	**IS**	8,4	7.89	0.104076293	0.071088282	*vs* HD
**K**	18	4.47	**0.001050578**	**1.03792x10^{-6}**	*vs* IS	**K**	16	5.48	**0.003602233**	0.248146391	*vs* IS
**Nuclear Cyclin-D1**											
**NM**	5	0.00				**NM**	5	0.00			
**IBD**	5	0.00	0.373900966			**IBD**	12	4.47	**0.024896163**		
**LD**	5	0.00	0.373900966	0.814902011	*vs* IBD	**LD**	5	0.00	0.373900966	**0.0249018**	*vs* IBD
**HD**	5	0.00	0.373900966	0.411406737	*vs* LD	**HD**	8	2.74	0.070483997	0.070483999	*vs* LD
**IS**	7	2.74	0.177807808	0.177750897	*vs* HD	**IS**	13	6.71	**0.056000000**	0.089009343	*vs* HD
**K**	17	6.71	**0.01613009**	**0.021742978**	*vs* IS	**K**	20	0.00	**1.89731x10^{-15}**	0.080097572	*vs* IS

Supplementary Table S3. Wnt pathway targeting compounds in clinical trials

Compound	Target	Modality	Tumor Type	Clinical Trials Identifier	Phase	Sponsor
**LGK974**	Porcupine (essential for Wnt protein secretion and function)	Small molecule	Melanoma; Breast Neoplasms; Lobular Carcinoma; Triple-negative Breast Cancer; Pancreatic Adenocarcinoma	NCT01351103	Phase 1	Novartis Pharmaceuticals
**BHQ880**	DKK-1	Antibody	Multiple Myeloma	NCT00741377<br>NCT01337752<br>NCT01302886	Phase 1<br>Phase 2<br>Phase 2	Novartis Pharmaceuticals
**DKN-01**	DKK-1	Antibody	Multiple Myeloma; Solid Tumors; Non-Small Cell Lung Cancer	NCT01457417<br>NCT01711671	Phase 1<br>Phases 1/2	Dekkun Corporation
**OMP-18R5**	Fzd receptors	Antibody	Solid Tumors	NCT01345201<br>NCT01957007	Phase 1<br>Phase 1	OncoMed Pharmaceuticals, Inc
**OMP-54F28**	Wnt ligands	Antibody	Solid Tumors	NCT01608867	Phase 1	OncoMed Pharmaceuticals, Inc<br>Collaborator: Bayer
**PRI-724**	β-catenin/CBP antagonist	Small molecule	Myeloid Leukemia; Pancreatic Adenocarcinoma; Solid tumor	NCT01606579<br>NCT01764477<br>NCT01302405	Phases 1/2<br>Phase 1<br>Phase 1	Prism Pharma Co., Ltd<br>Collaborator: inVentiv Health Clinical

Detailed information available on http://clinicaltrials.gov
